# Bioengineering a Human Dermal Equivalent Using Induced Pluripotent Stem Cell-Derived Fibroblasts to Support the Formation of a Full-Thickness Skin Construct

**DOI:** 10.3390/cells14141044

**Published:** 2025-07-08

**Authors:** Lucy Smith, David Bunton, Michael Finch, Stefan Przyborski

**Affiliations:** 1 Department of Biosciences, Durham University, Durham DH1 3LE, UK; 2 Reprocell Europe Ltd., Thomson Pavilion, Acre Road, Glasgow G20 0XA, UK; david.bunton@reprocell.com (D.B.); michael.finch@reprocell.com (M.F.)

**Keywords:** induced pluripotent stem cells, tissue engineering, in vitro models, fibroblasts, human skin equivalents, 3D cell culture

## Abstract

In vitro tissue models offer a flexible complementary study system for use alongside in vivo human tissue samples. Achieving accurate in vitro models relies on combining appropriate scaffolds, growth factors and cell populations to recreate human tissue complexity. Balancing a consistent cell supply with the creation of healthy tissue models can be challenging; established cell lines are often cancerous, with altered cellular function compared to healthy populations, and primary cells require repeated isolation, with associated batch-to-batch variation. Pluripotent stem cell-derived populations offer a consistent supply, as well as the ability to model disease phenotypes through cell reprogramming using patient-derived cells. In this study, we have used an induced pluripotent stem cell-derived fibroblast population to develop a dermal equivalent model. These cells form a consistent tissue construct with a structure and composition similar to primary fibroblast controls, which are able to support an overlying epidermis. The resultant full-thickness skin model demonstrates the expression of various key skin-related markers, correctly localised within the organised epidermis, notably improving on previous models of a similar nature. Providing proof of concept using an established in vitro protocol, this study paves the way for future work developing consistent, customised, full-thickness human skin equivalents using iPSC-derived populations.

## 1. Introduction

While using fresh human tissue samples is the most accurate research technique for understanding human health and disease, it is well appreciated that complementary study systems can offer greater insight when answering current biological questions. Each methodology has its own strengths and limitations; careful selection and combination provide increased value to scientific studies, where tissue-specific effects can be studied within in vivo samples alongside the detailed investigation of biological mechanisms in more flexible in vitro systems. Complex and physiologically accurate in vitro tissue models have revolutionised human health research [1], allowing for the routine performance of highly controlled, customised studies using bioengineered tissue equivalents. The reproducible nature of in vitro tissue models allows for continuous and high-throughput experiments due to their ‘on demand’ availability, overcoming sourcing issues that can occasionally be associated with using human samples and providing the additional benefit of stable transport between laboratories. The ability to maintain in vitro tissue models in the long term permits extended or dynamic studies which are not always possible with fresh tissue samples, expanding the range of experiments which can be performed. The myriad technologies now available allow researchers to select the optimal bioengineered tissue system to suit their research techniques and questions, in order to arrive closer to the biological answers they are searching for.

There are three general factors to consider when developing an in vitro tissue model: scaffold/structure, cytokines/growth factors and cell population. Scaffolds are the physical structure on which the tissue model will form, providing mechanical and even chemical cues dependent on the technique used. Growth factors may be incorporated into scaffolds or added to culture media to influence cellular behaviour through direction towards proliferation or differentiation. The cell population used in any in vitro model is key to model success and thus can be the most difficult aspect to optimise, balancing the need for healthy, functional cell populations with a sustainable and consistent supply. Primary cell populations are the most desirable to use, and while some cell populations can be easily isolated from tissues in usable quantities, such as fibroblasts, some demand highly specialist handling during and following isolation. Due to this, repeated cell isolations are required to continue experimental work, such as for respiratory alveolar epithelial cells [2]. The significant benefits of using primary cell populations to develop tissue models, such as being able to create models of disease, are counterbalanced by the batch-to-batch variation between different donors, at times requiring culture protocols to be reoptimised to support the behaviour of the new batch [3,4]. While cancerous or transformed cell lines offer more consistency and a continuous cell supply, they often possess additional genetic mutations which impact cellular function, leading to in vitro models which do not mimic the full functionality of the original tissue and may ultimately provide inaccurate results [5,6]. Pluripotent stem cells (PSCs) are an attractive alternative cell source for in vitro models, which, when used effectively, can encompass the benefits of both primary cells and their cancerous counterparts. Although PSCs require differentiation towards a specific cell identity, many differentiation protocols are now well optimised and established, providing the ability to create unlimited, functional cells of any tissue type, including from patient donors.

In the regenerative medicine field, the focus now is on utilising PSC populations to develop complex in vitro models representative of in vivo human tissues. Much progress has been made in specific tissue types; cerebral organoids are well established and utilised widely in the neurobiology field [7,8]. Tissues which contain a range of distinct cell types have more complex differentiation requirements, such as skin, and thus need more sustained input to develop the cell populations and resultant tissue models. While the value of PSCs as a resource cannot be overstated, the other elements involved in building an in vitro tissue model must not be overlooked. Many studies have shown that ensuring correct spatial arrangement of cells in three dimensions is critical to maintaining proper reception of physical and chemical cues and ultimately cell function [9,10]. A small number of induced PSC (iPSC)-derived skin models have been previously developed, but some lack the natural anatomical structure seen in human skin or use exogenous protein matrices which impact cellular structure and function [11,12,13,14,15,16].

Previously, we have established an in vitro human skin equivalent using primary human cells and a physical porous scaffold, allowing cells to achieve the proper spatial arrangement for normal function, resulting in a skin construct which is highly similar to human skin [17]. While we have utilised a range of other primary or already-differentiated cell types in this model, such as ageing cells [18,19], melanocytes [20] and sensory neurones [21], in this study, we have pursued an alternative approach, using iPSC-derived fibroblasts to build the dermal compartment. We show that the iPSC-derived fibroblasts form a consistent dermal model, which is highly similar to the primary human dermal fibroblast model in its structure and extracellular matrix composition. We also show that primary keratinocytes can form a recognisable epidermis when seeded onto the dermal structure, which shows structural similarity with the full primary cell skin equivalent and human native skin samples. This study demonstrates the significant potential of this technology for developing physiologically and anatomically relevant human skin equivalents using components derived from pluripotent cell populations.

## 2. Materials and Methods

### 2.1. Derivation of Human iPSC-Derived Fibroblasts

Human iPSC-derived fibroblasts were generated from StemRNA™ Human iPSCs lot number 771-3G (Reprocell Inc, Yokohama, Japan). Differentiation into fibroblasts was carried out by Reprocell Inc, according to a previously published protocol [13], and the fibroblast phenotype was confirmed by flow cytometry for the following markers: vimentin (ab185030, Abcam, Cambridge, UK), CD10, CD44, CD73 and CD90 (555375, 555479, 555479, 550257, BD Biosciences, Wokingham, UK). Differentiated cells were frozen down in Cellbanker 1 plus (Zenogen Pharma, Fukushima, Japan) until use.

### 2.2. Routine Maintenance of Cultured Cells

Neonatal human dermal fibroblasts (HDFns, lot #1366434, Thermo Fisher Scientific, Loughborough, UK) were revived and banked in Synthafreeze™ (Thermo Fisher Scientific) according to the manufacturer’s instructions. Cells were maintained in Human Basal Fibroblast Expansion medium (Thermo Fisher Scientific) supplemented with Low Serum Growth Supplement (LSGS, Thermo Fisher Scientific). Cells were passaged enzymatically using Trypsin/EDTA and were used in 3D models between passages 4 and 6.

iPSC-derived fibroblast cultures were provided by Reprocell Inc. Cells were revived and banked in Cell Banker 2 (AMSBio, Abingdon, UK) according to the manufacturer’s instructions. Cells were maintained in 0.1% gelatin (Stem Cell Technologies, Cambridge, UK)-coated 6-well plates (Greiner Bio One, Stonehouse, UK) in Dulbecco’s modified Eagle’s Medium with High Glucose and GlutaMax™ (Thermo Fisher Scientific) supplemented with Foetal Bovine Serum (FBS, Thermo Fisher Scientific), 1% Non-Essential Amino Acids (NEAAs, Thermo Fisher Scientific) and 10 ng/mL basic fibroblast growth factor (bFGF, Peprotech, Thermo Fisher Scientific). Cells were passaged using Accutase (Stem Cell Technologies) and used in 3D models up to a population doubling level of 12.

Neonatal human epidermal keratinocytes (HEKn, lot numbers #2286578 and #2286109) were revived and banked in Synthefreeze™ according to the manufacturer’s instructions. Cells were maintained in Epilife (Thermo Fisher Scientific) supplemented with Human Keratinocyte Growth Supplement (HKGS, Thermo Fisher Scientific). Cells were used in full-thickness models at passage 3.

### 2.3. Formation of 3D Skin Models

The methodology for generating bioengineered human skin was adapted from Roger et al., 2019 [17], with significant adjustments made to the formation of the dermis by iPSC-derived fibroblasts. The procedure to generate these full-thickness skin models was performed over two steps, which are described as follows:

1) Insert preparation and dermal model generation.

On the day of dermal model seeding, Alvetex^®^ 12-well scaffolds (Reprocell Europe Ltd., Sedgefield, UK) were prepared for use according to the manufacturer’s protocol as follows: Inserts were initially placed into 6-well plates and soaked in 70% ethanol to render them hydrophilic, before washing in Phosphate-Buffered Saline (PBS) and adding a small amount of dermal media to the insert to prevent it from drying out. Following this, fibroblast populations, which had been cultured to 70–80% confluency, were prepared for seeding to form dermal models.

Primary HDFn cultures were washed with PBS and detached from cultureware using Trypsin/EDTA, with the reaction being neutralised using Trypsin Neutraliser solution (Thermo Fisher Scientific). HDFns were then counted using the Trypan Blue exclusion assay to determine cell viability. The medium was removed from the prepared inserts, and 0.5 M viable cells were seeded on top of each 12-well insert in approximately 200 µL medium in a dropwise manner, ensuring even coverage across the surface. Inserts were then incubated at 37 °C for at least 2 h to allow cells to attach to the scaffold, before topping up each well with 10 mL of dermal model media consisting of Human Basal Fibroblast Expansion medium, LSGS, 1% Penicillin/Streptomycin, 100 µg/mL ascorbic acid and 5 ng/mL Transforming Growth Factor-β (TGF-β).

iPSC-derived fibroblasts were washed with PBS before being detached from cultureware using Accutase, with maintenance media, as described above, added to neutralise the reaction. iPSC-derived fibroblasts were then counted using the Trypan Blue exclusion assay to determine cell viability, and seeded onto the inserts as described above, with 0.5 M per 12-well insert in a dropwise manner, using the dermal model media. In selected experiments where the DMEM formulation was used for iPSC-derived fibroblast dermal models, media consisted of the following: DMEM, 10% FBS, 1% NEAA, 1% Penicillin/Streptomycin, 100 µg/mL ascorbic acid and 5 ng/mL TGF-β.

Dermal models were subsequently cultured at 37 °C and 5% CO_2_ for up to 28 days, with full media changes every 3–4 days.

2) Full-thickness model formation.

To form full-thickness skin equivalents, 28-day-old dermal models were used. Primary HEKns, which had been grown to 70–80% confluency, were detached from cultureware using Trypsin/EDTA and the reaction neutralised using Trypsin Neutraliser solution. HEKns were counted using the Trypan Blue exclusion assay to determine cell viability. Just before HEKn seeding, dermal models were moved to fresh 6-well plates, with any media remaining on the model carefully aspirated, avoiding touching the dermal model. Then, 1.3 M viable HEKns were seeded onto each dermal model at approximately 200 µL in a dropwise manner, to ensure even coverage across the surface. Models were then incubated at 37 °C for at least 2 h to allow cells to attach to the scaffold, before topping up each well with 10 mL of submerged culture media consisting of the following: Epilife, HKGS, 1% Penicillin/Streptomycin, 10 ng/mL keratinocyte growth factor, 100 µg/mL ascorbic acid and 140 µM calcium chloride. Models were then cultured at 37 °C and 5% CO_2_ for 48 h without interference.

Following this, models were moved to the air–liquid interface using the Alvetex^®^ Well Insert holder and Deep Petri Dish (Reprocell Europe Ltd.). The medium was aspirated from full-thickness model inserts, carefully ensuring that all liquid was removed from the top of the models without disturbing the keratinocyte layer. Models were then placed into the Well Insert holder on the middle setting, with 3 models in each Deep Petri Dish. A total of 35 mL of air–liquid interface media was carefully added to the Deep Petri Dish area below the models, which is sufficient to touch the base of each model, creating the air–liquid interface condition. Air–liquid interface media consisted of Epilife, HKGS, 1% Penicillin/Streptomycin, 10 ng/mL keratinocyte growth factor, 100 µg/mL ascorbic acid and 1.64 mM calcium chloride. Full-thickness models were then cultured at 37 °C and 5% CO_2_ for a further 14 days, with full media changes every 3–4 days.

### 2.4. Tissue Model Processing

Upon harvesting, models were washed twice in PBS before fixing in 4% paraformaldehyde (Thermo Fisher Scientific) for 2 h at room temperature. Models were dehydrated through a series of ethanols up to 100%, before being incubated in Histoclear II (National Diagnostics, Atlanta, GA, USA) for 30 min. Samples were then incubated in a 1:1 ratio of Histoclear II/molten wax (Cell Path, Powys, UK) at 60 °C for at least 30 min, or until the mixture had completely melted. Samples were then incubated in pure molten wax for 60 min at 60 °C, before embedding in wax in plastic moulds. Samples were sectioned at 5 µm on a Leica RM2125 RT rotary microtome and mounted onto SuperFrost charged microscope slides (Thermo Fisher Scientific).

### 2.5. Histology

Haematoxylin and Eosin staining was performed as subsequently described. Slides were deparaffinised in Histoclear I (National Diagnostics, Scientific Laboratory Supplies, Nottingham, UK) for 20 min, before rehydrating in 100%, 95% and 70% ethanols and distilled water. Slides were stained in Mayer’s haematoxylin solution (Merck, Dorset, UK) for 5 min, before washing for 30 s in distilled water. Slides were dehydrated in 70% ethanol for 30 s, before being incubated in alkaline ethanol for 30 s to develop the characteristic blue of the nuclei. Slides were washed in 70% ethanol and further dehydrated in 95% ethanol for 30 s each, before staining in Eosin Y (Merck) in 95% ethanol for 30 s. Slides were then washed twice in 95% ethanol for 10 s each, before dehydration in 100% ethanol for 15 s and 30 s. Slides were cleared in Histoclear I for 5 min, before clearing again in fresh Histoclear I. Slides were mounted onto glass coverslips in Histomount or Omnimount (National Diagnostics).

### 2.6. Immunofluorescence

Slides were deparaffinised in Histoclear I for 20 min, before rehydration in 100% and 70% ethanols and washing in PBS. Antigen retrieval was performed by incubating slides in pH 6 citrate buffer at 95 °C for 20 min and allowing slides to cool to room temperature. Blocking was performed at room temperature for 60 min, using 20% normal calf serum (Thermo Fisher Scientific) in 0.4% Triton-X-100 in PBS. Primary antibodies were added to the slides (see Table 1) and incubated either at room temperature for 60 min or at 4 °C overnight. Following this, slides were washed 3 times in PBS for 10 min each, before appropriate secondary antibodies and Hoescht 33342 (Thermo Fisher Scientific) were added, and incubated for 60 min at room temperature. Slides were washed another 3 times in PBS for 10 min each, before mounting on glass coverslips with Vectashield^®^ Antifade Mounting Medium (Vector Labs, Newark, NJ, USA). Slides were stored at 4 °C away from light until imaging was performed using a Zeiss 800 confocal microscope with Airyscan or the Zeiss Axioskop 40 fluorescent microscope (Carl Zeiss Microscopy Ltd., Cambridge, UK).

### 2.7. Collagen Assay

A total collagen hydroxyproline assay (QuickZyme Biosciences, Leiden, The Netherlands) was performed according to the manufacturer’s instructions; 3 mm punch biopsy samples were taken from individual 3D models and processed using the protocol provided in the kit.

### 2.8. Fibronectin Assay

A fibronectin ELISA (R&D Systems, Abingdon, UK) was performed according to the manufacturer’s instructions; 3 mm punch biopsy samples were taken from individual 3D models and processed according to the protocol provided in the kit.

### 2.9. Human Skin Samples

Healthy human skin samples were obtained from surgical residual sources with full ethical consent from the patient according to Reprocell tissue protocol TPS-011-UK, under REC reference number 22/WS/0007. Human skin samples were fixed in 10% neutral buffered formalin and dehydrated through a series of ethanols, 70%, 80%, 90%, 95% and 100%, before being embedded in wax as described above.

## 3. Results

### 3.1. iPSC-Derived Dermal Fibroblasts Are Similar in Expression Profile to HDFns and Grow Successfully in a Primary Fibroblast Culture Medium

**Figure 1:**
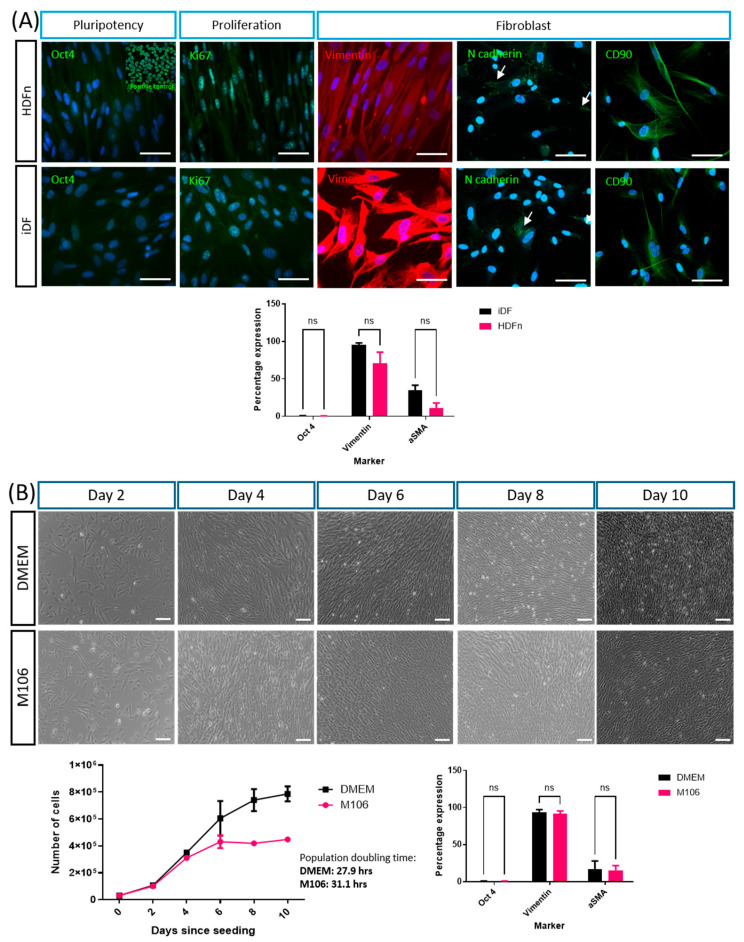
iPSC-derived fibroblasts show a similar expression profile to primary human dermal fibroblasts and grow in culture medium compatible for bioengineering skin equivalents: (**A**) iPSC-derived fibroblasts display the expected expression profile, similar to that observed in HDFns. Pluripotency marker Oct4 is not expressed (insert image shows positive control for Oct4 staining in cultured human pluripotent stem cells (TERA2.SP12)), but positive expression of Ki67 indicates proliferative capacity. iPSC-derived fibroblasts express a range of fibroblast markers, including vimentin, N cadherin and CD90. (**B**) When cultured in the defined medium used for primary cell culture and skin model generation, iPSC-derived fibroblasts survive and proliferate, increasing in number over the growth period. In the two media types, the expression of the selected markers as analysed by flow cytometry is found to be the same. All immunofluorescent images are representative. For growth curves, population doubling time was calculated using the slope between days 2 and 4 of culture. In all graphs, data is from 3 independent experiments, displaying the mean ± SEM. Statistical significance was determined using multiple unpaired *t*-tests with Welch’s correlation to assume individual variance in each group, and Holm–Sidak multiple comparisons conducted at threshold *p* (0.05). ns = not significant. Scale bars = 100 µm.

iPSC-derived fibroblasts (iDFs) were initially cultured in the recommended medium (DMEM with described supplements) and their morphology and expression profile compared to primary HDFns cultured in the recommended medium (Human Fibroblast Basal Expansion Medium supplemented with Low Serum Growth supplement (referred to as M106 herein)). iDFs proliferated and showed an expression profile of multiple markers similar to that of HDFns (Figure 1A). iDFs showed the absence of staining for pluripotency marker Oct4, indicating the loss of pluripotency. Positive staining for Ki67 indicated the proliferative capacity of the population. Positive staining for fibroblast markers vimentin, N cadherin and CD90 demonstrated the fibroblast phenotype in the population. Flow cytometry data confirmed the lack of Oct4 expression, high vimentin expression and relatively low α-smooth muscle actin expression, as seen in HDFns. Importantly, flow cytometry analysis confirmed that this expression profile was maintained when iDFs were cultured in M106. Growth analysis demonstrated that iDFs were able to survive and proliferate in the M106 medium (Figure 1B). Phase-contrast imaging and cell counts indicated an increase in cell number over time and the formation of a viable cell population. The population doubling time for iDF cultures was calculated to be 31.1 h in the M106 medium, which is reduced compared to that in the DMEM-based medium, 27.9 h.

### 3.2. iPSC-Derived Fibroblasts Migrate into the Scaffold, Forming a Three-Dimensional Dermal Construct

**Figure 2:**
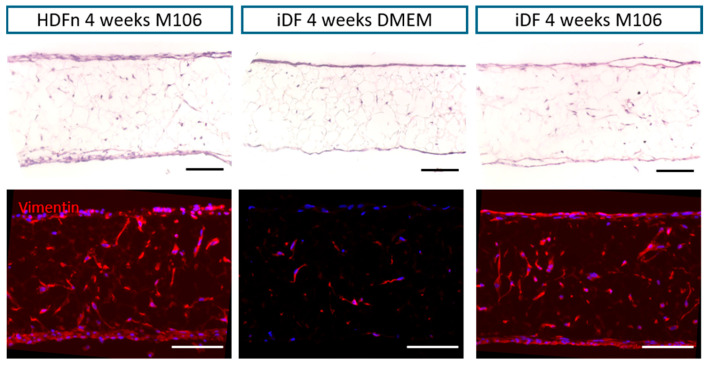
iPSC-derived fibroblasts integrate into the porous scaffold, forming a dermal construct, using the established protocol: Representative histological staining shows that iPSC-derived fibroblasts are able to enter and successfully proliferate throughout the scaffold, filling spaces to form a dermal model in the same way as the primary dermal fibroblasts. Vimentin staining highlights the cells within the scaffold. All immunofluorescent images are representative. Scale bars = 100 µm.

To initiate the bioengineering of the skin equivalent, iDFs were used to make dermal constructs, with the same cell number used to set up each model. Two media types were tested: DMEM- and M106-based media with Transforming Growth Factor-β (TGF-β) and ascorbic acid supplementation. iDFs migrated into the scaffold over the 4-week culture period, forming a three-dimensional dermal construct, with the layering of cells at the top and bottom of the polystyrene membrane, similar to the HDFn primary cell model (Figure 2). Immunofluorescent staining for fibroblast marker vimentin highlighted that the fibroblasts are present throughout the entire scaffold, demonstrating the successful filling of voids and pores. The formation of a dermal construct was achieved using both media types; thus, M106 was used for subsequent experiments to allow for direct comparison with the human primary dermal fibroblast control model.

### 3.3. iPSC-Derived Fibroblasts Showed Increasing Cell Number and Extracellular Matrix Deposition over Time

**Figure 3:**
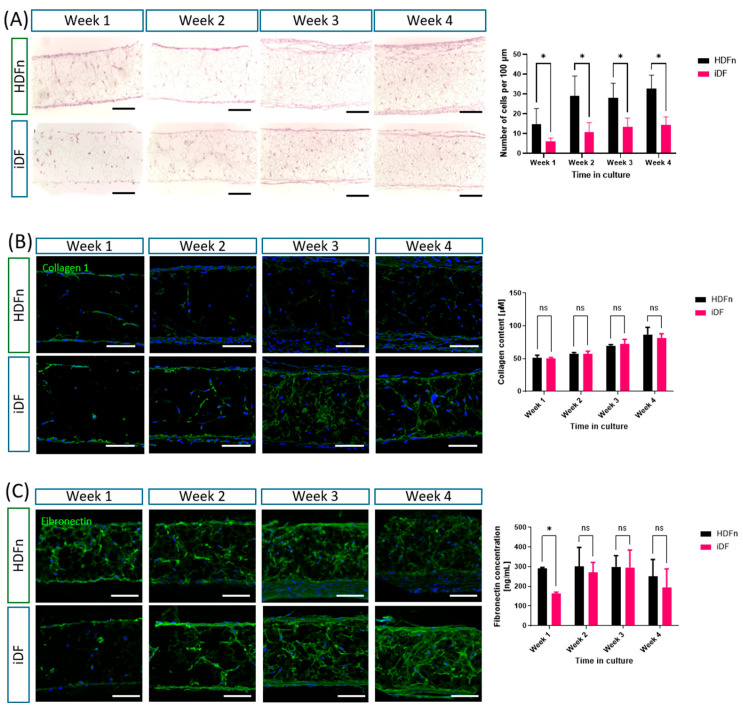
iPSC-derived fibroblasts proliferate in the porous scaffold and deposit extracellular matrix proteins over time in a similar way to primary human dermal fibroblasts: (**A**) More detailed experiments confirm that iPSC-derived fibroblasts progressively fill the scaffold over time, although cell numbers remain lower than those observed in primary HDFn models. (**B**) Evidence of collagen deposition can be seen in immunostaining for collagen 1 and accompanying quantitative data for total collagen content within punch biopsy samples from the dermal models, which show no significant difference compared to HDFn models and a steady increase over time. (**C**) Fibronectin deposition can also be detected through immunostaining images and quantitative data from a fibronectin ELISA, again showing little difference from the quantity found in the HDFn dermal models. All immunofluorescent images are representative. In all graphs, data is from 2 to 3 independent experiments, displaying the mean ± SEM, with 3 samples analysed per experiment. Statistical significance was determined using multiple unpaired *t*-tests with Welch’s correlation to assume individual variance in each group, and Holm–Sidak multiple comparisons conducted at threshold *p* (0.05). ns = not significant; * = *p* < 0.05. Scale bars = 100 µm.

Detailed parallel studies were conducted to compare the formation of the iDF dermal model to the HDFn primary cell dermal model over the 4-week formation period, using the same experimental conditions (cell number and media composition). Histology and immunofluorescent staining data in Figure 3A show the development of the dermal models during culture, with iDFs showing a steady increase in cell number as seen in the HDFn models and characteristic layering at the top and bottom of the scaffolds. Interestingly, the cell number in iDF dermal models is found to be consistently around 2-fold lower than the cell number in time-matched HDFn dermal models, and fewer layers can be noted on the outer edges of the scaffold. Collagen 1 is an abundant and common extracellular matrix component of the human dermis, which is found in the iDF dermal models, as in HDFn models, as seen in Figure 3B. The protein shows an even distribution within the iDF model in immunostaining images and was detected at similar levels to HDFn models using a quantitative hydroxyproline-based assay. Fibronectin is another key extracellular matrix protein which is secreted by iDFs and detected through qualitative immunostaining at increasing levels within the dermal model, as seen in Figure 3C. A qualitative ELISA shows no significant difference in the amount found in the iDF dermal models compared to the HDFn dermal models, despite there being an approximate 2-fold difference in the numbers of cells with the dermal compartment.

### 3.4. Full-Thickness Human Skin Models Can Be Created on iPSC-Derived Fibroblast Dermal Models, Expressing Expected Markers of Differentiation and Proliferation in a Correctly Localised Manner as Seen in In Vivo Human Skin

**Figure 4:**
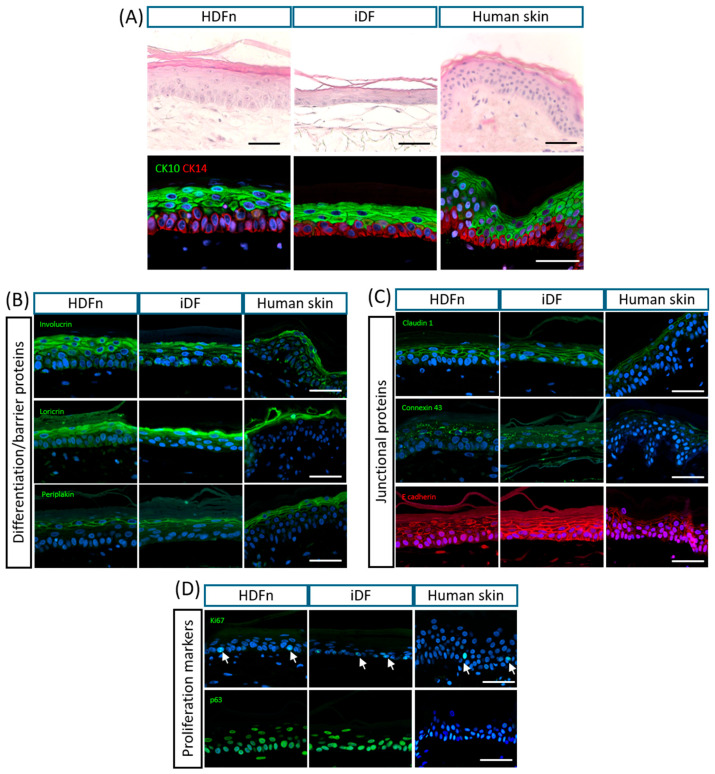
iPSC-derived fibroblasts support the formation of a full-thickness skin model which shows similarities to in vitro and in vivo human skin: (**A**) iPSC fibroblast dermal models support the formation of full-thickness skin models using primary human keratinocytes, which resemble the overall structure of human skin as seen in the primary cell model and human skin samples. Correct localisation of cytokeratins 14 and 10 to the basal and suprabasal layers, respectively, shows that stratification and differentiation have occurred. (**B**) iPSC-based full-thickness models show the expression and correct localisation of barrier proteins involucrin, loricrin and periplakin, indicating that the dermal model can support cornification and barrier function. (**C**) The distribution of junctional proteins in the epidermis of the iPSC fibroblast dermal model matches that seen in the primary cell model and the human skin samples. (**D**) Proliferation markers Ki67 and p63 indicate the proliferative capacity of the basal cells in the epidermis, providing evidence that the iPSC dermal model can support the population. All immunofluorescent images are representative. Scale bars = 50 µm.

In order to test the capabilities of the iPSC-derived dermal model to support the formation and maintenance of an epidermis, primary human keratinocytes were seeded onto the iDF dermal model as described in the established protocol to form a full-thickness skin model. Figure 4 directly compares primary HDFn control models and iPSC-derived models with human native skin samples. In Figure 4A, H&E-stained images show the reproducible skin equivalent derived completely from primary cell populations, with a fully differentiated and stratified epidermis, mimicking the structure observed in human skin. The iDF model formed a recognisable epidermis on top of the dermal model, complete with polarised basal cells and a stratum corneum, although the model overall appeared thinner than the HDFn control. Immunostaining for cytokeratins 10 and 14 demonstrated the presence of distinct suprabasal and basal layers, respectively, indicating that the epidermis has successfully differentiated and polarised. Positive staining for barrier proteins involucrin, loricrin and periplakin in the iDF models was comparable to that seen in the HDFn controls and human skin samples (Figure 4B). This provided further evidence that the epidermis had undergone sequential differentiation and possessed barrier function, with these proteins localised to the uppermost layers of the structure as expected. The barrier integrity of the epidermis in the iDF models was confirmed by positive staining for a range of junctional proteins, as shown in Figure 4C, including E-cadherin, Claudin 1 and Connexin 43. The maintenance of the proliferative capacity of the basal layer was confirmed in all samples by positive staining for general proliferation marker Ki67 and epidermal basal stem cell marker p63 (Figure 4D).

## 4. Discussion

The use of fresh tissue samples or primary cells is considered the most desirable and accurate way to conduct human biological research; however, a combination of factors can restrict feasibility. These include the limited viability window during which experiments can be performed, the requirement for specialist equipment and careful monitoring, a complex legal and ethical framework surrounding the use of human samples and the simple fact that demand often outstrips supply. The use of complementary systems enhances scientific studies, with each bringing its own strengths in answering biological questions. In vitro tissue models can offer many of the benefits of human tissue, particularly when using primary cells and ensuring that the cellular microenvironment contains the correct spatial, physical and chemical cues to develop and maintain a complex tissue construct. However, the limited nature of primary cells creates the need to use multiple donor batches, resulting in biological variation which can impact model performance. Additionally, the inability to successfully isolate and maintain some cell populations prohibits their study. While transformed cells offer a potential option for consistent cell supplies, their inherent differences often prevent them from truly recapitulating their primary counterparts.

In this study, we have used iPSC-derived dermal fibroblasts to create full-thickness human skin equivalents which were compared to skin models created using primary dermal fibroblasts. We examined the growth and synthesis of the extracellular matrix within iPSC-derived dermal models compared to the primary cell controls, before using primary human keratinocytes to build a full-thickness skin equivalent. In-depth characterisation was then performed to assess the structure of the full-thickness models created using a dermal compartment derived either from iPSC-derived fibroblasts or from primary fibroblasts in comparison to native human skin tissue.

The initial characterisation of the iDFs aimed to ensure that fibroblast identity had been maintained on transfer to a different laboratory and to explore their phenotype when cultured in the same defined culture media used for the primary fibroblasts. The absence of immunostaining for the pluripotency regulator Oct4 contributed to confirming the differentiated state and absence of the stem cell phenotype. Positive immunostaining for fibroblast-associated markers—cytoskeletal protein vimentin [14], junctional protein N cadherin [22] and membrane glycoprotein CD90 [23]—helped confirm fibroblast identity. Flow cytometry data further confirmed the expected expression patterns for Oct4 and vimentin data for the fibroblast phenotype. The relatively low level of alpha smooth muscle actin expression was consistent with the fact that the fibroblasts remained in an unstimulated state. Growth curves compared alternative media compositions to ensure that iDFs were capable of proliferating in the M106 medium and that the phenotype was maintained. The small increase in population doubling time likely reflects the difference the in nutritional composition of the M106 defined medium, a phenomenon also noted in the primary cells used in these experiments [24,25]. Population growth was reduced and plateaued around day 6 when iDFs were cultured in the defined media, as opposed to the continued increase observed in the recommended DMEM formulation. It should be noted that the DMEM formulation contains basic fibroblast growth factor (also known as FGF-2), a potent stimulator of fibroblast growth [26], which enhances proliferation. Flow cytometry demonstrated no significant difference in the expression of selected markers when iDFs were cultured in the two media types.

Initial experiments compared the ability of iPSC-derived fibroblasts and primary dermal fibroblasts to integrate with the scaffold, again using the alternative media types. Previous work has shown that a range of fibroblast types can enter and populate the porous scaffold successfully [18,19,27]. The data herein demonstrate that the same successful outcome was replicated using iDFs, indicating the possibility of creating a full-thickness skin model using these cells in this established system.

Subsequent experiments focused on a week-by-week comparison of dermal models formed using iDFs and primary human dermal fibroblasts, investigating cell number and extracellular matrix deposition, when cultured in the M106 medium. The histology data confirmed the sustained increase in iDFs inside and on the surface of the scaffold membrane over time. It was also noted that there are generally fewer iDFs in the models compared to constructs containing primary fibroblasts. Quantitative analysis confirmed that the number of cells in the iDF models was consistently around half of that observed in time-matched primary cell dermal models. This is unexpected given the fact that the same number of cells were seeded on the porous scaffold at the beginning of dermal culture. The reduced iDF population within the scaffold did not impact extracellular matrix (ECM) synthesis. Quantitative analysis showed no significant difference in the deposition of key ECM proteins collagen 1 and fibronectin between the two populations. iPSC-derived fibroblast populations have previously been shown to synthesise the ECM, but this was often either in conventional 2D cell culture or in combination with exogenous protein matrices, which could influence results [11,14]. A recent study has also noted the enhanced ability of iPSC-derived fibroblasts to generate ECM proteins in an alternative defined medium, a beneficial property for generating in vitro skin models without using animal products [11]. The unique nature of the system reported herein permits close monitoring of endogenous ECM synthesis in response to changing culture conditions coupled with a consistent anatomical 3D structure and direct comparison to primary cell controls. Sufficient evidence was collected in these dermal model studies to undertake pilot studies assessing the ability of the iDF dermal models to maintain an epidermis.

Primary human keratinocytes were seeded onto both the iDF and primary cell control models to produce a full-thickness skin model. This parallels other studies which also used this stepwise approach to examine the capabilities of the dermal model [13]. However, the final step of introducing iPSC-derived keratinocytes in addition to iPSC-derived fibroblasts to form full-thickness skin models appeared to cause issues in other studies. What was common to these was the variability in the formation of the epidermis in the full-thickness skin equivalent. Many such full-thickness skin models reportedly showed poor structure and organisation, and at times were unrecognisable as a stratified, differentiated epidermis [11,13,14,16]. A study which also incorporated iPSC-derived melanocytes into an iPSC-derived full-thickness skin model does show a more recognisable phenotype [12], with an observable stratum corneum, yet the structural organisation of the epidermis is still variable. While the use of iDFs resulted in a partly thinner epidermis, the iDF dermal model reported herein was able to support the formation of a recognisable and organised epidermis comparable in overall structure to models created using primary dermal fibroblasts and native tissue architecture. The epidermal tissue has a polarised basal layer of cells, as evidenced by distinct staining for cytokeratins 10 and 14, and a differentiated stratum corneum on the surface, consistent with the structure of the primary cell control and the human skin samples. Further characterisation using a range of skin markers confirmed their correct spatial localisation, including key barrier and junctional proteins. These data indicate that epidermal stratification and differentiation processes had successfully occurred in skin equivalents built on iPSC-derived cells.

Whilst we have successfully demonstrated the ability of human iPSC-derived fibroblasts to produce a dermal equivalent comprising an endogenously produced ECM, it is also important to recognise the limitations and scope for improvement. For example, our data indicate the formation of human ECM components, but they are not in any way exhaustive. Additional work is required to assess the ECM composition in a more in-depth manner, making comparisons to the control and native human skin with more extensive analytical capability such as by applying proteomic approaches. Similarly, analysis of the secretome of the dermal compartment would also demonstrate comparability to the native dermis. Indeed, we have previously shown that such signalling is an important aspect in the development of HSEs (18). The HSE developed in this study is composed of only two cell types, one of which is a primary cell population. Logical next steps to address this would include using iPSC-derived keratinocytes to form the full-thickness HSE, to determine whether the improved epidermal organisation can be maintained within a model produced exclusively from iPSC-derived cells. This would also further elucidate the role of the stem cell-derived dermis in the formation and support of the epidermis. The relatively simple nature of our stem cell-derived HSE model lends itself well to the generation and addition of other integral skin cell populations, such as melanocytes or immune cells. For example, we have successfully created human skin equivalents using primary pigmented and immune cells based on similar platform technology [20,28]. The incorporation of skin appendages, such as hair follicles or sebaceous glands, is much more sophisticated to reproduce but is an ultimate goal as technology advances. Collectively, such research tools would expand the potential uses of HSEs as well as provide more accurate insights into biological questions. The applications of HSE technology are wide ranging, from fundamental skin biology work to generating patient-derived HSEs to study inherited skin conditions or the cutaneous component of genetic diseases. Personalised medicine studies would also be possible following further development of stem cell-derived HSEs, allowing for the selection of efficient therapeutics; this may be especially useful for major clinical issues such as chronic skin ulceration, whereby patients can be screened for a range of potential therapies and any underlying wound features explored. Whilst there are many exciting possibilities for such technology, it is essential that the methodology to construct such models is developed in incremental steps, which are checked and validated. Indeed, the current study exemplifies a step in this direction, adopting this approach.

In summary, these data provide compelling evidence that iPSC-derived fibroblasts are capable of producing an endogenous extracellular matrix within a scaffold membrane designed to support the bioengineering of a human skin construct. The dermal equivalent generated contained ECM proteins characteristic of native skin tissue that are suitable to support the growth and differentiation of the epidermis. This study paves the way for future work developing a human full-thickness skin equivalent produced entirely from iPSC-derived cell populations using this approach. This approach would also be valuable to support other epithelial tissue models that would benefit from such stromal interactions.

## Figures and Tables

**Table 1:** Primary and secondary antibodies used in immunofluorescent staining.

Antibody	Species	Supplier	Product Code	Dilution
Cytokeratin 10	Rabbit	Abcam (Cambridge, UK)	ab76318	1:200
Cytokeratin 14	Mouse	Abcam	ab7800	1:200
Loricrin	Rabbit	Abcam	ab85679	1:100
Involucrin	Rabbit	Abcam	ab53112	1:100
Periplakin	Rabbit	Abcam	ab131269	1:100
Claudin-1	Rabbit	Abcam	ab15098	1:100
Connexin 43	Rabbit	Proteintech (Manchester, UK)	26980-1-AP	1:100
E-cadherin	Mouse	BD Biosciences (Wokingham, UK)	610181	1:200
Collagen IV	Rabbit	Abcam	ab6586	1:100
Collagen I	Rabbit	Southern Biotech (Birmingham, USA)	1310-01	1:100
Collagen III	Rabbit	Abcam	ab7778	1:100
Ki67	Rabbit	Abcam	ab16667	1:200
p63	Rabbit	Abcam	ab124762	1:100
Fibronectin	Rabbit	Abcam	ab23750	1:100
Donkey Anti-Rabbit IgG Alexa Fluor^®^ 488	Donkey	Thermo Fisher Scientific	10544773	1:1000
Donkey Anti-Mouse IgG Alexa Fluor^®^ 488	Donkey	Thermo Fisher Scientific	10348022	1:1000
Donkey Anti-Rabbit IgG Alexa Fluor^®^ 594	Donkey	Thermo Fisher Scientific	10424752	1:1000
Donkey Anti-Mouse IgG Alexa Fluor^®^ 594	Donkey	Thermo Fisher Scientific	10798994	1:1000
Donkey Anti-Goat Cross-Adsorbed DyLight 488	Donkey	Thermo Fisher Scientific	13477237	1:1000

## Data Availability

Data are available from the authors on request.

## Abbreviations

The following abbreviations are used in this manuscript:

3D	Three-dimensional
bFGF	Basic fibroblast growth factor
DMEM	Dulbecco’s modified Eagle’s medium
ECM	Extracellular matrix
FBS	Foetal bovine serum
HDFns	Human neonatal dermal fibroblasts
HEKns	Human neonatal epidermal keratinocytes
HKGS	Human keratinocyte growth supplement
iDFs	Induced pluripotent stem cell-derived fibroblasts
iPSC	Induced pluripotent stem cell
LSGS	Low serum growth supplement
M106	Medium 106 or human fibroblast expansion basal medium
NEAAs	Non-essential amino acids
PBS	Phosphate-buffered saline
PSCs	Pluripotent stem cells
TGF-β	Transforming growth factor-β
